# Genetic and Biological Characteristics of Duck-Origin H4N6 Avian Influenza Virus Isolated in China in 2022

**DOI:** 10.3390/v16020207

**Published:** 2024-01-30

**Authors:** Tian Li, Chuankuo Zhao, Yuxin Guo, Jinze Dong, Fanshu Du, Yong Zhou, Sicheng Shu, Yang Liu, Yachang Cheng, Zhiyong Cao, Qi Cao, Shuiping Shi, Yinhua Huang, Juan Pu, Litao Liu

**Affiliations:** 1 Key Laboratory for Prevention and Control of Avian Influenza and Other Major Poultry Diseases, Ministry of Agriculture and Rural Affairs, College of Veterinary Medicine, China Agricultural University, Beijing 100193, China; litiansweet7@163.com (T.L.); zck0523@163.com (C.Z.); yuuuxin_guo@163.com (Y.G.); dongjinze@cau.edu.cn (J.D.); dudfs97@163.com (F.D.); zhouyong@cau.edu.cn (Y.Z.); sicheng_shu@163.com (S.S.); pujuan@cau.edu.cn (J.P.); 2 State Key Laboratory of Biocontrol, School of Ecology, Sun Yat-Sen University, Guangzhou 510275, China; liuy353@mail.sysu.edu.cn (Y.L.); chengych3@mail.sysu.edu.cn (Y.C.); 3 Duchang County Aquaculture and Animal Husbandry Industry Development Center, Jiujiang 332600, China; 18770269096@139.com; 4 Duchang County Yangfeng Township Government, Jiujiang 332600, China; 18711702705@163.com; 5 Duchang County Migratory Bird Nature Reserve Administration, Jiujiang 332600, China; 5237661dchn@163.com; 6 State Key Laboratory for Agrobiotechnology, College of Biology Sciences, China Agricultural University, Beijing 100193, China; cauhyh@cau.edu.cn

**Keywords:** avian influenza virus, H4N6, evolution, pathogenicity, transmission

## Abstract

The interaction between migratory birds and domestic waterfowl facilitates viral co-infections, leading to viral reassortment and the emergence of novel viruses. In 2022, samples were collected from duck farms around Poyang Lake in Jiangxi Province, China, which is located within the East Asia–Australasia flyway. Three strains of H4N6 avian influenza virus (AIV) were isolated. Genetic and phylogenetic analyses showed that the isolated H4N6 avian influenza viruses (AIVs) belonged to new genotypes, G23 and G24. All isolated strains demonstrated dual receptor binding properties. Additionally, the isolated strains were able to replicate efficiently not only in avian cells but also in mammalian cells. Furthermore, the H4N6 AIV isolates could infect chickens, with viral replication detected in the lungs and extrapulmonary organs, and could transmit within chicken flocks through contact, with viral shedding detected only in oropharyngeal swabs from chickens in the contact group. Notably, the H4N6 AIV could infect mice without prior adaptation and replicate in the lungs with high viral titers, suggesting that it is a potential threat to humans. In conclusion, this study provides valuable insight into the characteristics of H4N6 strains currently circulating in China.

## 1. Introduction

Wild birds and waterfowl are recognized as natural hosts and expansive reservoirs of avian influenza viruses (AIVs), frequently harboring the virus asymptomatically [1]. The interaction between migratory birds and domestic waterfowl can facilitate co-infection by different subtypes of AIVs, leading to viral reassortment and the emergence of novel viruses [2]. Novel influenza viruses usually circulate in chickens first to establish widespread prevalence and transmission, and undergo significant adaptive mutations before they can effectively breach host barriers to infect mammals or humans, such as H7N9 [3,4] and H3N8 [5]. In the last two decades, the incidence of AIVs crossing species boundaries to infect humans has risen [6]. This emphasizes the necessity of the surveillance of AIVs in wild birds and waterfowl.

The H4 AIV is prevalent among global populations of wild birds and waterfowl [1,7,8]. Additionally, the virus has also been naturally identified in chickens [8,9] and mammals, including pigs [10,11] and seals [12], indicating a wide host range. Furthermore, serological studies involving poultry farm workers in the United States and Lebanon demonstrated specific antibodies against H4 AIV in humans [13,14]. Currently, H4 AIVs are widely circulating in China, undergoing complex and frequent reassortment, resulting in various unique combinations of H4 subtypes [8,15]. Therefore, monitoring, biological evaluation, and risk assessment of H4 AIVs, which are endemic at the interface of migratory birds and waterfowl, are important for obtaining an early warning of its public health threats posed by outbreaks of H4 AIV in humans [16].

Poyang Lake in Jiangxi Province, China, is one of the most important wintering grounds for waterbirds along the “East Asia–Australasian” migratory flyway and supports the intersection of wild birds and local waterfowl [17]. In this study, three strains of H4N6 viruses were isolated from samples collected from duck farms around Poyang Lake. The viral genes were systematically analyzed, and the biological characteristics of the viruses were further evaluated to enhance the understanding of H4N6 AIVs.

## 2. Materials and Methods

### 2.1. Cells

Human lung epithelial (A549), chicken embryo fibroblast (CEF), and Madin–Darby canine kidney (MDCK) cells were maintained in Dulbecco’s modified Eagle’s medium (DMEM; Life Technologies, Foster City, CA, USA) supplemented with 10% fetal bovine serum (FBS; Life Technologies, Foster City, CA, USA), 100 units/mL of penicillin, and 100 µg/mL of streptomycin.

### 2.2. Virus Isolation

A total of 129 swab samples were collected from 19 duck farms in the Poyang Lake area of Jiangxi Province in 2022. Each sample was suspended in an antibiotic solution in phosphate-buffered saline (PBS) containing 1000 U/mL penicillin and 1000 U/mL streptomycin, and then centrifuged at 13,800× *g* for 10 min. The filtered supernatants were inoculated into the allantoic cavity of 9-day-old specific pathogen-free (SPF) embryonated chicken eggs and incubated at 35 °C. Allantoic fluid from the incubated eggs was harvested 48 h after inoculation. A hemagglutination (HA) assay was conducted with 1% packed chicken red blood cells as described previously [18].

### 2.3. Genome Sequencing and Phylogenetic Analysis

Viral RNA was directly extracted from infected allantoic fluid by using a QIAamp Viral RNA Mini Kit (Qiagen, Hilden, Germany). Multiplex reverse transcription–PCR amplification was performed on RNA samples using a PrimeScript™ II 1st Strand cDNA Synthesis Kit (Takara Bio, Kyoto, Japan) and influenza universal primers [19]: 0.5 μL Uni12/Inf1 (5′-GGGGGGAGCAAAAGCAGG-3′), 0.5 μL Uni12/Inf3 (5′-GGGGGAGCGAAAGCAGG-3′), and 1 μL Uni13/Inf1 (5′-CGGGTTATTAG-TAGAAACAAGG-3′). The cDNA samples were then sent to the Institute of Microbiology, Chinese Academy of Sciences, for next-generation sequencing. The resulting sequencing data of the viral strains were aligned using NCBI BLAST (http://blast.ncbi.nlm.nih.gov/Blast.cgi, accessed on 28 April 2022) sequence comparison.

For genetic comparison, reference sequences were obtained from the GISAID (https://gisaid.org/, accessed on 10 August 2023) and GenBank (https://www.ncbi.nlm.nih.gov/genbank/, accessed on 10 August 2023) databases. Phylogenetic trees were constructed by using the neighbor-joining method implemented in MEGA 7.0 [20], with 1000 bootstrap replicates. A distinct phylogenetic lineage and bootstrap support values ≥80% indicated a common origin for group delineation. The sequence data were compiled by using the SEQMAN program (DNASTAR, Madison, WI, USA) based on the reference sequences, and molecular markers in each segment were identified by using the MegAlign program (DNASTAR, Madison, WI, USA).

### 2.4. Classification of Genotype

Virus genotypes were defined by gene phylogeny as previously described [21]. A genotype was defined when the phylogenetic lineages of the eight genes resulted in a unique gene grouping or constellation. Genotypes were divided into two types: (1) major genotypes that were detected for 2 years or more; and (2) transient genotypes that were only detected within a single year [22]. To better understand the evolutionary relationship of the major genotypes of H4N6 influenza viruses in China, we used a similar genotyping method used for H5N1 influenza viruses [22] to classify the major genotypes into different series based on their differences in gene constellation. Specifically, we compared a genotype to the genotypes that emerged before it. (1) If this genotype differed from the previous genotypes in the lineage by just one or two internal gene segments, we classified it as belonging to the same series. (2) If a genotype was different from all the previous genotypes in the lineage for three or more internal gene segments, we defined this genotype as a new series. The numbers of genotype names in each series were arranged according to the time of emergence for each genotype. The genotype that emerged earlier was given the former number, e.g., G1. For the genotypes that emerged in the same year, the genotype that was most similar to the previous genotype gene constellation was designated as the former number. In this study, genotype classification was carried out exclusively for reference sequences and isolate sequences.

### 2.5. Receptor Binding Analysis

Receptor binding specificity was analyzed with a solid-phase binding assay as previous described [23]. Briefly, plates (15500, Thermo Fisher Scientific, Waltham, MA, USA) were coated with serial dilutions of α-2,3 (3′SLN: Neu5Acα2-3Galβ1-4Glcβ-sp4-PAA-biotin) and α-2,6 glycans (6′ SLN: Neu5Acα2-6Galβ1-4GlcNAcβ-sp3-PAA-biotin) (GlycoNZ, Auckland, New Zealand) overnight at 4 °C. The glycan solution was then removed, and the plates were blocked by 5% skim milk solution, washed by phosphate-buffered saline containing 0.05% Tween (PBST), and incubated with a solution containing 2^6^ HA units of each influenza virus type. The plates were incubated at 4 °C for 10 h. After washing, the plates were incubated with chicken serum against H4N6 AIV. Subsequently, the plates were washed and incubated with HRP-conjugated goat anti-chicken antibody (1:10,000; AE072224, Bioss, Beijing, China). Finally, the plates were washed and incubated with 3,3′,5,5′-Tetramethylbenzidine (TMB) two-component substrate solution (Soliabio, Beijing, China) for 10 min at room temperature. The reaction was stopped with 0.5 M H_2_SO_4_, and the absorbance was measured at 450 nm. Three independent experiments were performed.

### 2.6. Virus Replication Kinetics In Vitro

Virus titers were determined by TCID_50_ in MDCK cells. The titrated viruses were used to infect A549 cells and CEF cells at a dose of MOI 0.01. Infection was carried out in DMEM containing 0.6 μg TPCK and supplemented with 1000 U/mL penicillin and 1000 U/mL streptomycin. The cells were then incubated at 37 °C in a cell culture incubator, and the medium was replaced after 1 h post infection (hpi). Cell culture supernatants (100 μL) were collected at 12, 24, 36, 48, 60, and 72 hpi. Three independent experiments were performed.

### 2.7. Chicken Infection Studies

Six-week-old SPF chickens were utilized to access the replication and pathogenicity of the H4N6 AIV in chickens. Six chickens were intranasally inoculated with 10^6^ EID_50_ of A/duck/Jiangxi/F011502/2022 (H4N6) (JX02) or PBS in 0.2 mL volume. Three chickens from each group were euthanized at 3 days post infection (dpi), and harderian gland, nasal turbinate, lung, trachea, liver, spleen, pancreas, kidney, duodenum, and bursa of Fabricius were collected for virus titration by EID_50_. For histopathological examination, the lung and nasal turbinate samples from infected chickens were collected at 3 dpi, fixed in 4% buffered paraformaldehyde, embedded in paraffin, sectioned into 5 μm slices, stained with hematoxylin and eosin (HE), and analyzed using light microscopy. For immunohistochemistry (IHC), an anti-influenza A antibody against the nucleoprotein (ab20343, Abcam, Beijing, China) was used for IHC staining as described previously [24].

### 2.8. Chicken Transmission Experiments

In contact transmission experiments, three chickens were intranasally inoculated with 10^6^ EID_50_ of JX02 in 0.2 mL volume. The next day, three inoculated chickens were individually paired by co-housing with a naïve chicken. Oropharyngeal and cloacal swabs were collected at 1, 3, 5, 7, 9, 11, and 13 dpi from the inoculated group and the contact group for detection of virus shedding by EID_50_.

### 2.9. Mouse Infection Studies

Five- to six-week-old female BALB/c mice were used to investigate the replication and pathogenicity of H4N6 AIV. Mice were anesthetized using Zoletil 50 (Zoletil; Virbac SA, Carros, France) by intramuscular injection, and mice were intranasally inoculated with 10^6^ TCID_50_ of JX02 or PBS in 50 μL volume. Three mice from each group were euthanized on 3 and 5 dpi, and lung, nasal turbinate, brain, liver, spleen, kidney, and duodenum were collected for virus titration in MDCK cells by TCID_50_. The remaining five mice in each group were monitored daily for weight loss and survival for 14 days. HE and IHC staining were conducted as described previously.

### 2.10. Statistical Analysis

Experimental groups were statistically compared by using analysis of variance (ANOVA). A *p* value of <0.05 was considered to indicate a statistically significant difference.

## 3. Results

### 3.1. Prevalence of H4N6 AIVs in Wild Birds and Waterfowl

**Figure 1:**
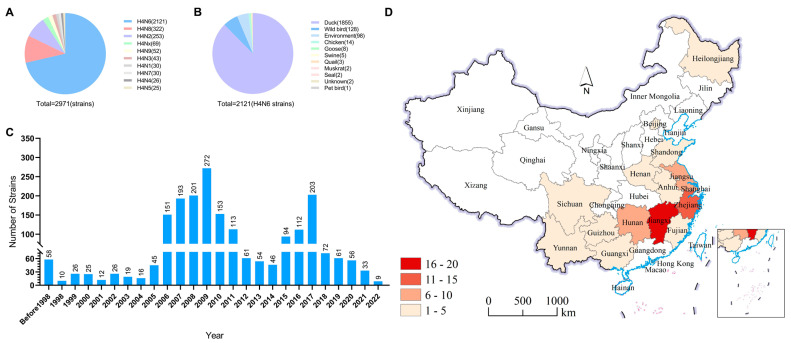
Prevalence of H4N6 AIVs. (**A**) HA and NA combinations of H4Nx strains in the database. (**B**) Summarized analysis of the hosts of H4N6 AIVs. (**C**) Number of H4N6 AIVs detected in wild birds from 1956 to 2023. (**D**) Distribution of H4N6 AIVs detected in China. All the public data in GenBank and GISAID.

To better understand the ecology and epidemiology of H4N6 AIVs, we mapped the globally circulated H4N6 AIVs in different hosts. To date, nine surface combinations of H4 and NA, namely, H4N1, H4N2, H4N3, H4N4, H4N5, H4N6, H4N7, H4N8, and H4N9, have been identified in animals, and a total of 2974 HA sequences of H4Nx (N1–N9) viruses obtained from birds and mammals are available in databases (GenBank and GISAID, updated to 10 August 2023) (Figure 1A). Among these nine identified subtypes, H4N6 (2121 HA sequences) and H4N8 (322 HA sequences) viruses are the predominant subtypes, and they have been detected in multiple animals. A total of 2121 H4N6 viral HA sequences were available in the databases, and these viruses were mainly detected in duck (1815 strains) and wild birds (128 strains). Only 14 H4N6 sequences from chickens were present in the GenBank and GISAID databases. H4N6 viruses have occasionally been found in geese, pigs, quails, muskrats, seals, and pet birds (Figure 1B). The H4N6 virus was first detected in 1956 and has circulated in animals for more than half a century. Moreover, there was a peak in the detection of H4N6 during the period between 2006 and 2011 (Figure 1C). A total of 71 H4N6 AIVs were detected in wild and domestic birds in 14 provinces in China (including the isolations detected in this study), with the highest number detected in Jiangxi Province (16 of 71), where they were isolated mainly from ducks. The number of cases detected in Jiangxi’s neighboring provinces, such as Zhejiang (11 of 71) and Hunan (7 of 71), was also high (Figure 1D).

The above results indicate that H4N6 AIVs have a wide range of hosts, but their main hosts are ducks and wild birds, with a small number of infections in chickens and mammals. In China, most of the provinces where H4N6 viruses have been isolated are located along the East Asia–Australia migratory bird migration routes, with Jiangxi province having the highest isolation rate, which can be considered a hotspot for the isolation of H4N6 AIVs.

### 3.2. Isolation of H4N6 AIVs from Duck Farms around Poyang Lake

Three H4N6 AIVs were isolated from 129 samples collected from 19 duck farms, with one from ground soil in the environment and two from oropharyngeal swabs of ducks (Table S1). The isolated viruses were then sequenced and named as A/environment/Jiangxi/F0114/2022 (H4N6) (referred to as JX14), A/duck/Jiangxi/F011501/2022 (H4N6) (referred to as JX01), and A/duck/Jiangxi/F011502/2022 (H4N6) (referred to as JX02).

### 3.3. Phylogenetic Analysis of H4N6 AIVs

**Figure 2:**
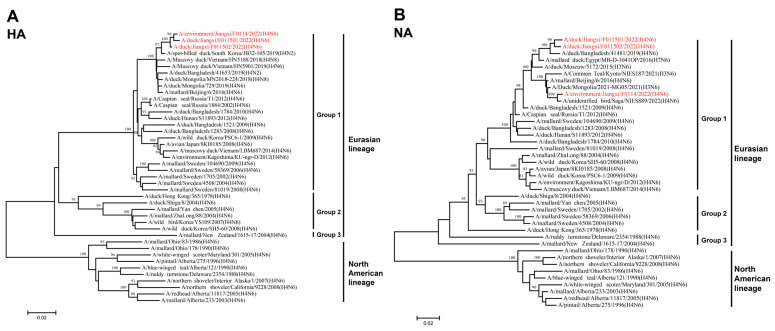
Phylogenetic tree of the (**A**) HA and (**B**) NA genes for the isolated viral strains. The tree was generated by the neighbor-joining method and bootstrapped with 1000 replicated using the MEGA 7.0 software. Neighbor-joining bootstrap values of ≥80 were shown at the major nodes of the phylogenetic trees. Phylogenetic trees were based on the comparison of nucleotide sequences of the H4 AIVs isolated in this study to reference AIV sequences published in GenBank. Isolates are highlighted in red. The scale bar represents the distance unit between sequence pairs.

To better understand the genetic evolutionary relationships of the three isolated H4N6 AIVs, a phylogenetic analysis was conducted on their eight gene segments, resulting in the construction of a phylogenetic tree. Overall, the eight genes of JX01, JX02, and JX14 belonged to the Eurasian lineage. The HA genes from the Eurasian lineage exhibited nucleotide homology ranging from 81.37% to 98.82% and could be further divided into three groups, with the highest similarity to an H4N2 subtype AIV isolated from spot-billed ducks in South Korea in 2019 (Figure 2A; Table S2). Similarly, the NA gene phylogeny could also be divided into three groups, with nucleotide homologies ranging from 84.36% to 98.78%. JX01 and JX02 clustered closely with an H4N6 subtype AIV isolated from ducks in Bangladesh in 2019, while JX14 clustered with an H4N6 subtype AIV isolated from poultry in Japan in 2022 (Figure 2B; Table S2).

**Figure 3:**
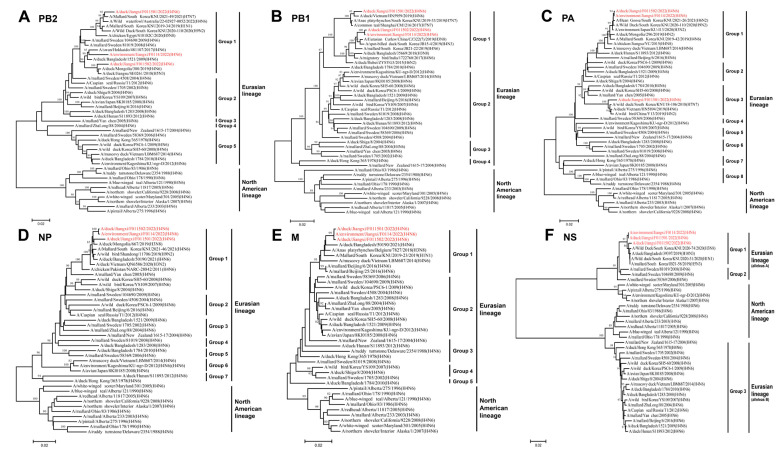
Phylogenetic tree of the six internal genes for the isolated viral strains. The six genes are (**A**) PB2, (**B**) PB1, (**C**) PA, (**D**) NP, (**E**) M, and (**F**) NS. The tree was generated by the neighbor-joining method and bootstrapped with 1000 replicates using the MEGA 7.0 software. Phylogenetic trees were based on the comparison of nucleotide sequences of the H4 AIV isolated in this study to reference AIV sequences published in GenBank. Isolates are highlighted in red. The scale bar represents the distance unit between sequence pairs.

Among the internal genes, the nucleotide homologies of the PB2 gene ranged from 83.56% to 98.46%. Notably, JX01, JX02, and JX14 exhibited the highest nucleotide similarity to A/Mallard/South Korea/KUN2021-46/2021 (H7N7), A/duck/Mongolia/380/2019 (H4N6), and A/swan/Hokkaido/481107/2017 (H4N6), respectively (Figure 3A; Table S2). The nucleotide homologies of the PB1 gene ranged from 87.69% to 98.85%. JX01 shared the highest nucleotide similarity with A/duck/Vietnam/HN5959/2019 (H4N6), JX02 shared the highest nucleotide similarity with A/Eurasian Curlew/China/CZ322/2019 (H3N8), while JX14 clustered with JX02 (Figure 3B; Table S2). For the PA gene, nucleotide homologies ranged from 87.87% to 99.72%. JX02 and JX14 clustered with A/bean geese/South Korea/KUN2021-26/2021 (H6N2), while JX01 clustered with A/wild duck/South Korea/KUN18-106/2018 (H7N7) (Figure 3C; Table S2). The NP gene exhibited nucleotide homologies ranging from 88.78% to 99.13%, and they clustered with A/duck/Mongolia/667/2019 (H3N8) (Figure 3D; Table S2). The homology of M genes was remarkably high, that is, 92.87–99.88%, and shared the highest nucleotide similarity to A/duck/Bangladesh/50190/2021 (H4N6) (Figure 3E; Table S2). The nucleotide homology of NS gene ranged from 77.05 to 99.41% and shared the highest nucleotide similarity to A/wild duck/South Korea/KUN2020-74/2020 (H3N8) (Figure 3F; Table S2).

Based on the phylogenetic diversity, we classified the 22 representative Eurasian lineage H4N6 AIVs together with three isolated H4N6 AIVs in this study into 24 genotypes (Table 1). The genotypes of isolated strains were G23 (JX14 and JX02) and G24 (JX01), differing from the genotypes of previously isolated strains. These results indicate that novel reassortments of H4N6 AIVs have occurred among waterfowl in China.

### 3.4. Molecular Characteristics of the Isolated H4N6 AIVs

In order to further clarify the replication ability, pathogenicity, and mammalian adaptation of the isolated virus, we analyzed key molecular markers. All three isolated virus strains had the amino acid motif ^324^PEKASR↓GLF (H3 numbering, which is used throughout this work) at the HA cleavage site, with only one basic amino acid, which was consistent with the molecular characteristics of low pathogenicity avian influenza virus (LPAIV). The HA receptor binding site (RBS) consists of four structural elements: the 130-loop, 150-loop, 190-helix, and 220-loop. A T160A mutation was identified at the 150-loop in the HA gene of all three viruses, which has been demonstrated to facilitate H5N1 and H9N2 AIVs in binding to human-type receptors [25]. Therefore, we can tentatively speculate that the isolates may have acquired the ability to bind to α-2,6 sialic acid-linked receptors, enabling them to infect both poultry and mammals. However, the remaining sites were highly conserved, such as 190E, 225G, 226Q, and 228G. The HA protein also had five conserved potential glycosylation sites: ^6^NYT^8^, ^22^NGT^24^, ^165^NLT^167^, ^296^NIS^298^, and ^483^NGT^485^. There were no deletions in the stem region of the NA protein, and no resistance mutation H274Y (N2 numbering) was observed. However, nine potential glycosylation sites were identified on the NA protein: ^51^NET^53^, ^54^ NST^56^, ^62^NNT^64^, ^67^NFT^69^, ^70^NIT^72^, ^86^NLT^88^, ^146^NGT^148^, ^201^NAS^203^, and ^402^NWS^404^. An increase in glycosylation sites may result in altered viral antigenicity.

The mammalian host adaptive mutations PB2-E627K/V and D701N were not detected in any of the H4N6 isolates. However, The PB2 protein of the isolated strains had the amino acid mutation I504V. Previous reverse genetic studies have found that the mutations in the PB2 protein could promote influenza virus replication and enhance the pathogenicity of influenza viruses [26]. Furthermore, no mutations were found in PB1 and PA. Additionally, no amantadine resistance mutations (e.g., S31N) in the M2 protein or deletions in the NS1 protein were observed in isolated H4N6 AIVs. However, mutations I43M and T215A were found in the M1 protein, and mutations I106M and C138F were found in the NS1 protein. These imply that all viruses could exhibit increased virulence in mice [27] (Table 2).

### 3.5. Receptor Binding Properties of the Isolated H4N6 AIVs

**Figure 4:**
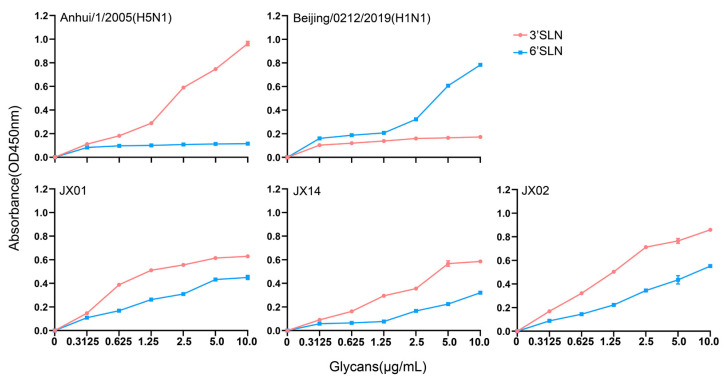
The sialic acid receptor binding characteristics of H4N6 AIVs. Direct binding of the viruses to sialylglicopolymers containing either α-2,3 sialic acid-linked (red) or α-2,6 sialic acid-linked (blue) were tested. Two viruses, A/Anhui/1/2005(H5N1) and A/Beijing/0212/2019(H1N1), that bind exclusively to α-2,3 and α-2,6 sialic acid-linked receptors, respectively, were used as controls. The data shown are the means from three replicates; the error bars indicate standard deviations.

The receptor binding preference of the HA protein plays a crucial role in cross-species transmission of influenza viruses. Typically, AIVs preferentially bind to α-2,3 sialic acid-linked receptors, while human influenza viruses prefer binding to α-2,6 sialic acid-linked receptors. Mutations in the receptor-binding domains of the HA protein may lead to alterations in viral receptor preferences, potentially impacting the virus’s host tropism and transmission capacity. Our previous analyses revealed the presence of a T160A mutation on the 150-loop of the HA protein that might alter the receptor binding properties of H4N6. Subsequently, we conducted solid-phase binding assays to verify the receptor preference of the isolated strains. The results showed that all three tested viruses could bind not only to the α-2,3 sialic acid-linked receptors but also to α-2,6 sialic acid-linked receptors, with a higher affinity for α-2,3 sialic acid-linked receptors. Among the three strains, JX02 exhibited the highest affinity for both types of receptors. This indicates that the isolated H4N6 AIVs have dual receptor binding properties and may possess the ability to infect both poultry and mammals (Figure 4).

### 3.6. Replication of H4N6 AIVs in Mammalian Cells and Avian Cells

**Figure 5:**
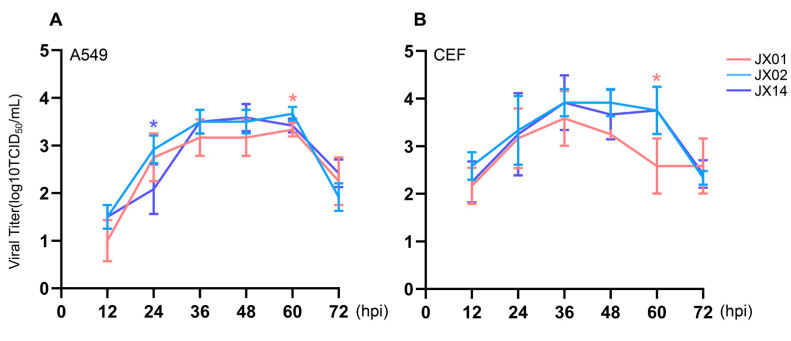
In vitro phenotype of H4N6 AIVs. Multistep growth curves of H4N6 AIVs in A549 (**A**) and CEF cells (**B**). Error bars represent standard deviations from the mean values (SD) for the independent experiments (*, *p* < 0.05).

To investigate the replication of H4N6 viruses in mammalian cells (A549 cells) and avian cells (CEF cells), we infected the cells with the virus at a dose of MOI = 0.01 and collected supernatants to determine the viral titers. All H4N6 AIVs were capable of replicating in both A549 cells and CEF cells. The replication of JX02 in A549 cells was significantly higher than that of JX14 at 24 hpi and JX01 at 60 hpi. Except for 24 hpi, the viral titer of JX14 was higher than that of JX01 at all other time points (Figure 5A). In CEF cells, the replication of JX02 was significantly higher than that of JX01 at 60 hpi, with no significant difference from JX14. Moreover, compared to JX01 and JX14, JX02 exhibited the best replication ability in both CEF and A549 cells (Figure 5B). Taken together, these findings indicate that the isolated H4N6 AIVs are capable of replicating in both human and avian cell lines.

### 3.7. Replication and Pathogenicity of H4N6 AIV in Chickens

**Figure 6:**
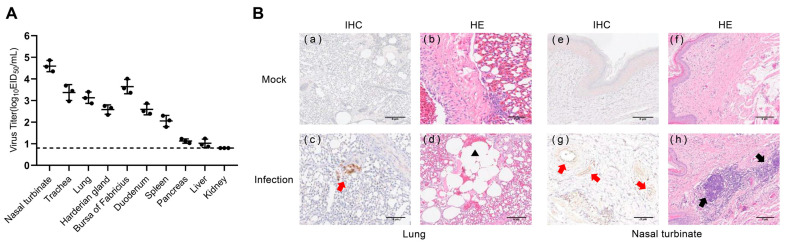
Virus titers in tissues and histopathological examination of infected chickens. (**A**) Chickens were inoculated intranasally with the viruses at a dose of 10^6^EID_50_. Three chickens from each group were euthanized at 3 dpi, and tissue samples (nasal turbinate, trachea, lung, harderian gland, bursa of Fabricius, duodenum, spleen, pancreas, liver, and kidney) were obtained for virus titration. The dotted line parallel to the X-axis represents the minimum detectable virus level. (**B**) Mock: (**a**,**b**) lungs and (**e**,**f**) nasal turbinate were harvested at 3 dpi from chickens inoculated intranasally with PBS; Infection: (**c**,**d**) lungs and (**g**,**h**) nasal turbinate were harvested at 3 dpi from chickens inoculated intranasally with 10^6^EID_50_ viruses. The red arrow indicates positive immunohistochemical reaction, the black arrow indicates inflammatory cell infiltration, and the black triangle represents pulmonary alveolar fusion.

To determine whether the isolated H4N6 AIVs could infect chickens, we infected three chickens with 10^6^ EID_50_ of JX02 in 0.2 mL volume and euthanized them at 3 dpi and collected lung and extrapulmonary organ samples for viral titration and histopathological detection. The virus was detected in 9 out of 10 chicken organs sampled, excluding the kidney. The highest viral titer was detected in the nasal turbinate (4.6 ± 0.3 log_10_EID_50_/mL) followed by the bursa of Fabricius (3.6 ± 0.3 log_10_EID_50_/mL), trachea (3.4 ± 0.4 log_10_EID_50_/mL), lung (3.1 ± 0.3 log_10_EID_50_/mL), duodenum (2.6 ± 0.3 log_10_EID_50_/mL), harderian gland (2.6 ± 0.2 log_10_EID_50_/mL), spleen (2.1 ± 0.3 log_10_EID_50_/mL), pancreas (1.1 ± 0.1 log_10_EID_50_/mL), and liver (1.0 ± 0.2 log_10_EID_50_/mL) (Figure 6A). Histopathological examination revealed inflammatory cell infiltration in the nasal turbinate, while the lung showed signs of pulmonary consolidation and fusion of alveoli, accompanied by mild congestion and hemorrhage. IHC staining of lung sections of chickens after infection showed the NP antigen, indicating that the H4N6 viruses replicated in chicken’s lungs (Figure 6B). These results indicate that the virus can infect chickens and replicate in their respiratory system.

### 3.8. Transmission of H4N6 AIV in Chickens

**Figure 7:**
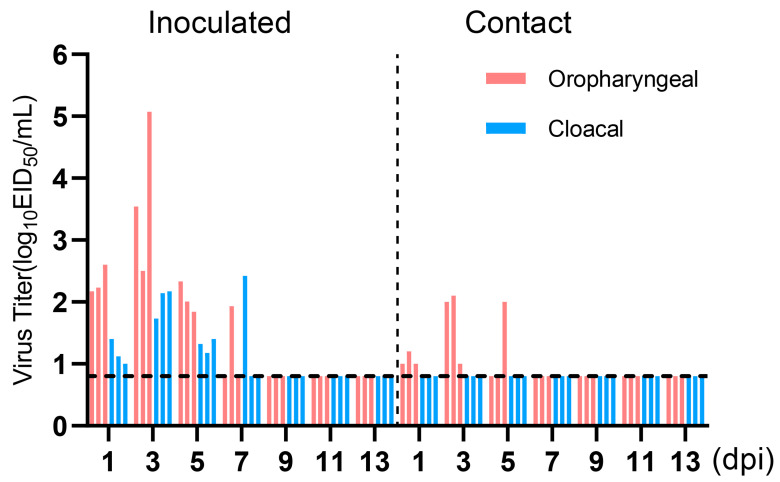
Virus titers in oropharyngeal and cloacal swabs of infected Chickens. Three chickens (6-week-old) were inoculated intranasally with the viruses at 10^6^EID_50_. Three chickens were introduced into the isolators later that day. Virus shedding in the respiratory and gastrointestinal tracts of both the inoculated group and the contact group was monitored using oropharyngeal and cloacal swabs, respectively, taken at 1, 3, 5, 7, 9, 11, and 13 dpi. Oropharyngeal swabs are labeled in red, cloacal swabs in blue. The dotted line parallel to the X-axis represents the minimum detectable virus level and samples from the inoculated group and the contact group are separated by a dashed line parallel to the Y-axis.

To evaluate the transmissibility of the H4N6 epidemic strain in chicken flocks, three chickens were infected with 10^6^ EID_50_ of JX02 in 0.2 mL volume, while three naïve chickens were co-housed with infected chickens at 1 dpi. Oropharyngeal and cloacal swabs were collected from all chickens every other day for 14 days and titrated by EID_50_. The results showed viral shedding in both the oropharyngeal and cloacal swabs of the inoculated group and the contact group. The viral load in oropharyngeal swabs of the inoculated group was significantly higher than in the cloacal swabs. Oropharyngeal shedding lasted for up to 7 days, with peak shedding occurring between 1 and 3 dpi, reaching a viral titer of 3.7 ± 1.3 log_10_EID_50_/mL. In the contact group, all three contact chickens had detectable virus in oropharyngeal swabs on 3 and 5 dpi, but the viral titers were low, measuring 1.7 ± 0.6 log_10_EID_50_/mL on 3 dpi and 1.6 ± 0.4 log_10_EID_50_/mL on 5 dpi. However, no virus shedding was observed in cloacal swabs (Figure 7). Overall, the results suggest that H4N6 has the potential to transmit but may not be able to sustain transmission and spread in chicken flocks.

### 3.9. Replication and Pathogenicity of H4N6 AIV in Mice

**Figure 8:**
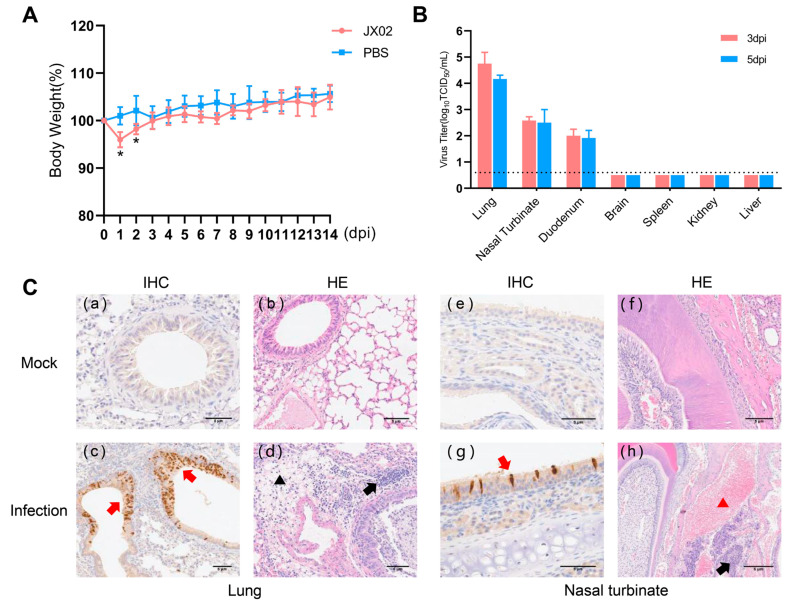
Weight changes, virus titers in tissues, and histopathological examination of infected mice. (**A**) Six-week-old female BALB/c mice were inoculated intranasally with 10^6^EID_50_/50 µL of virus. The body weights of inoculated mice were measured daily for 14 days and are represented as percentages of weight on the day of inoculation (day 0). The average for each group is shown. The red line represents the infected group, while the blue line represents the PBS control group. (**B**) Three mice from each group were euthanized at 3 and 5 dpi, and tissue samples (lung, nasal turbinate, duodenum, brain, kidney, spleen, and liver) were obtained for virus titration; 3 dpi are labeled in red, 5 dpi in blue. The dotted line parallel to the X-axis represents the minimum detectable virus level (*, *p* < 0.05). (**C**) Mock: (**a**,**b**) lungs and (**e**,**f**) nasal turbinate were harvested at 5 dpi from mice inoculated intranasally with PBS; Infection: (**c**,**d**) lungs and (**g**,**h**) nasal turbinate were harvested at 5 dpi from mice inoculated intranasally with 10^6^EID50 viruses. The red arrow indicates positive immunohistochemical reaction, the black arrow indicates inflammatory cell infiltration, the black triangle represents loose edema of connective tissue, and the red triangle represents congestion and hemorrhage.

To understand the replication and pathogenicity of the H4N6 epidemic strain in mammals, mice were infected with 10^6^ TCID_50_ of the JX02 strain in 50 μL volume, and it was observed that all mice survived. The infected mice showed a significant decrease in body weight at 1 and 2 dpi, followed by recovery (Figure 8A). The results of viral titration showed that although the viral load in each organ was slightly higher at 3 dpi than at 5 dpi, the difference was not statistically significant. Specifically, the JX02 virus exhibited higher replication levels in the lungs (4.8 ± 0.4 log_10_TCID_50_/mL; 4.2 ± 0.1 log_10_TCID_50_/mL), followed by the nasal turbinate (2.6 ± 0.1 log_10_TCID_50_/mL; 2.5 ± 0.5 log_10_TCID_50_/mL) and duodenum (2.0 ± 0.3 log_10_TCID_50_/mL; 1.9 ± 0.3 log_10_TCID_50_/mL). No virus was detected in the brain, liver, kidney, or spleen (Figure 8B). Consistently, IHC staining of lung and nasal turbinate sections of mice after infection confirmed the presence of the NP antigen, suggesting that H4N6 AIV can replicate in the mouse respiratory system. Histopathological examination revealed hemorrhage and an inflammatory cellular infiltrate in the nasal turbinate, while a certain degree of inflammatory cellular infiltration was predominant in the lungs (Figure 8C). In conclusion, the results showed that JX02 was able to replicate in infected mice, mainly in the respiratory system as well as in the digestive system, and these results indicate that H4N6 AIV possesses the ability to directly infect mammalian hosts without prior adaptation and highlights the potential cross-species infection risk of H4N6 AIV.

## 4. Discussion

The interaction between wild birds and domestic waterfowl can lead to the emergence of novel influenza viruses through reassortment, posing significant challenges to global poultry farming and public health [28]. In recent years, several LPAIVs, such as H3N8 and H10N3, have crossed species barriers to infect mammals and humans [2,5,29,30]. These incidents have raised concerns about the cross-species transmission of LPAIVs. The H4 subtype AIVs are widely prevalent among global wild birds and waterfowl, with detections reported during routine surveillance in countries across Asia, North America, and Europe [7,31,32]. Although H4 AIVs have been infrequently associated with clinical illness in poultry in recent years, various subtypes of H4 AIVs have cocirculated in central, eastern, and southern regions of China. Frequent genetic reassortment events have been observed among different H4 AIV subtypes and between H4 AIVs and other subtypes within the wild bird–waterfowl–poultry interface, contributing to the complex diversity of H4 AIV strains in Chinese waterfowl, particularly domestic ducks. The migratory behaviors of birds have exacerbated the occurrence of reassortment events [8,33,34].

In this study, we conducted genetic and phylogenetic analyses of three newly isolated duck-origin H4N6 AIVs and investigated their biological characteristics. Previous studies have found that multiple genotypes of H4 viruses are cocirculating in the live poultry markets of China [8], and the results of phylogenetic analysis and the genotypes in this study revealed that the three isolated viral strains belonged to novel genotypes, G23 and G24. The genetic sequences of various segments of the isolated strains shared high similarity with virus strains isolated from neighboring countries and regions around China, such as Bangladesh, Mongolia, Vietnam, South Korea, and Japan. Notably, these countries are located along the East Asia–Australasia flyway, a major migratory route for birds. Thus, we hypothesized that the genetic origin of the isolated strains was likely due to the introduction of different AIV subtypes through the migratory activities of birds, which then underwent reassortment with the AIVs carried by domestic waterfowl in China.

Molecular characterization analysis revealed that all three isolated strains had a T160A mutation in the HA gene associated with receptor binding properties. In addition to T160A, G228S, and Q226L play an important role in altering the receptor binding properties of H4 AIV [16]. Subsequently, we conducted an analysis of receptor binding characteristics. The results showed that the isolated strains could bind not only to α-2,3 sialic acid-linked receptors but also to α-2,6 sialic acid-linked receptors, indicating that H4N6 AIVs have the potential to break species barriers and initiate cross-species infections. Pathogenicity experiments on chickens revealed that an H4N6 virus isolate was able to replicate in infected chickens, although no significant clinical symptoms were observed. However, some studies have reported that H4N6 can cause weight loss in broiler chickens and soft-shelled eggs in laying hens [9]. Transmissibility experiments on chickens showed that the isolate had limited transmissibility and was not capable of becoming widespread in chicken flocks, and this characteristic was similar to the previously prevalent H4 subtype AIV, which was mainly found in waterfowl and migratory birds, and failed to adapt well to terrestrial poultry. Surprisingly, the isolate could infect mice directly without prior adaptation and exhibited robust replication in both the respiratory and digestive systems of mice. Other studies have found that some H4 strains have been found to be transmitted between guinea pigs by direct contact, and some can also transmit via respiratory droplet, albeit with limited efficiency [8].

In conclusion, we characterized the genetic evolution and biological properties of the H4N6 virus isolated from waterfowl in Poyang Lake, Jiangxi Province, China. Our study not only offers valuable insights into the prevalence of H4N6 AIVs in China but also highlights the importance of proactive monitoring of wild birds and domestic ducks. This monitoring aids in tracking the evolution of AIVs in waterfowl. It is crucial to monitor AIVs that may pose a threat to poultry or humans and to take appropriate control measures in time.

## Figures and Tables

**Table 1:** Genotypes of H4N6 AIVs.

Virus	Group of Each Gene Segment in the Phylogenetic Tree								Genotype
	HA	NA	PB2	PB1	PA	NP	M	NS	
A/duck/Hong Kong/365/1978	2	2	5	4	7	7	3	3	G1
A/mallard/Sweden/1705/2002	1	2	2	3	6	3	4	3	G2
A/mallard/Sweden/4508/2004	1	2	2	2	5	2	2	3	G3
A/mallard/New_Zealand/1615-17/2004	3	3	5	4	5	3	3	3	G4
A/duck/Shiga/8/2004	2	2	2	3	2	1	4	3	G5
A/mallard/ZhaLong/88/2004	2	1	4	3	7	3	2	3	G6
A/mallard/Yan chen/2005	2	2	3	3	3	1	2	3	G7
A/mallard/Sweden/58369/2006	1	2	5	2	4	5	2	2	G8
A/wild_bird/Korea/YS109/2007	2	1	2	2	5	1	4	3	G9
A/duck/Bangladesh/1283/2008	1	1	2	2	6	4	2	3	G10
A/avian/Japan/8KI0185/2008	1	1	2	2	7	6	2	3	G11
A/mallard/Sweden/81019/2008	1	1	1	2	6	4	3	2	G12
A/wild_duck/Korea/SH5-60/2008	1	1	5	2	3	1	2	3	G13
A/duck/Bangladesh/1521/2009	1	1	1	2	2	3	2	3	G14
A/wild_duck/Korea/PSC6-1/2009	1	1	5	2	2	2	2	3	G15
A/mallard/Sweden/104690/2009	1	1	1	2	2	2	2	2	G16
A/duck/Bangladesh/1784/2010	1	1	5	1	3	5	5	3	G17
A/environment/Kagoshima/KU-ngr-D/2012	1	1	5	2	4	6	2	3	G18
A/duck/Hunan/S11893/2012	1	1	2	2	1	7	3	3	G19
A/Caspian_seal/Russia/T1/2012	1	1	2	2	2	2	2	3	G20
A/muscovy_duck/Vietnam/LBM687/2014	1	1	5	2	1	6	1	3	G21
A/mallard/Beijing/6/2016	1	1	2	2	1	2	1	3	G22
A/environment/Jiangxi/F0114/2022	1	1	1	1	1	1	1	1	G23
A/duck/Jiangxi/F011501/2022	1	1	1	1	3	1	1	1	G24
A/duck/Jiangxi/F011502/2022	1	1	1	1	1	1	1	1	G23

**Table 2:** Molecular characteristics of the H4N6 viruses in this study.

Gene	Site	Function	JX14	JX01	JX02
HA (H3 numbering)	Cleavage site		^324^PEKASR↓GLF	^324^PEKASR↓GLF	^324^PEKASR↓GLF
	T160A	RBS positions altered receptor specificity	A	A	A
	E190D		E	E	E
	G225D		G	G	G
	Q226L		Q	Q	Q
	G228S		G	G	G
	Glycosylation sites		^6^NYT^8^, ^22^NGT^24^, ^165^NLT^167^, ^296^NIS^298^, ^483^NGT^485^	^6^NYT^8^, ^22^NGT^24^, ^165^NLT^167^, ^296^NIS^298^, ^483^NGT^485^	^6^NYT^8^, ^22^NGT^24^, ^165^NLT^167^, ^296^NIS^298^, ^483^NGT^485^
NA (N2 numbering)	H274Y	Antiviral resistance	H	H	H
	Glycosylation sites		^51^NET^53^, ^54^ NST^56^, ^62^NNT^64^, ^67^NFT^69^, ^70^NIT^72^, ^86^NLT^88^, ^146^NGT^148^, ^201^NAS^203^, ^402^NWS^404^	^51^NET^53^, ^54^ NST^56^, ^62^NNT^64^, ^67^NFT^69^, ^70^NIT^72^, ^86^NLT^88^, ^146^NGT^148^, ^201^NAS^203^, ^402^NWS^404^	^51^NET^53^, ^54^ NST^56^, ^62^NNT^64^, ^67^NFT^69^, ^70^NIT^72^, ^86^NLT^88^, ^146^NGT^148^, ^201^NAS^203^, ^402^NWS^404^
PB2	I504V	Enhanced polymerase activity and increased virulence in mice	V	V	V
	E627K/V	Mammalian adaptive mutations; enhanced polymerase activity and increased virulence in mice	E	E	E
	D701N		D	D	D
M1	I43M	Increased virulence in mice	M	M	M
	T215A		A	A	A
M2	S31N	Antiviral resistance	S	S	S
NS1	I106M	Increased virulence in mice	M	M	M
	C138F		F	F	F

## Data Availability

The genomic data presented in this study are available from GenBank (accession numbers: OR864242-OR OR864249, OR864250-OR864257, OR864258-OR864265).

## Supplementary Materials

The following supporting information can be downloaded at https://www.mdpi.com/article/10.3390/v16020207/s1: Table S1. Basic information on sampling; Table S2. The virus strains with the highest homology.
