# Supplementary material for: Genetic and Biological Characteristics of Duck-Origin H4N6 Avian Influenza Virus Isolated in China in 2022
Source: Viruses. 2024 Jan 30;16(2):207. doi: 10.3390/v16020207 (PMC10892581; doi:10.3390/v16020207)
Supplement: Supplementary file 1 [file viruses-16-00207-s001.zip › viruses-2783981-supplementary.pdf]

Table S1. Basic information on sampling.

Province	City	Number of farms	Breed	Collection date	Samples	Influenza isolates (O or C or E)*	Isolation rates (%)	Number of O or C or E samples positive for H4N6	Number of O or C or E samples positive for Other subtypes
Jiangxi	Nanchang	7	white-striped duck; white-striped green duck; mallard	Jan 2022	53	38, 0, 15(1)	1.9	1	0
	Jiujiang	12			60	38, 3, 19	0	0	0
	Shangrao	1			17	15(2), 0, 2	11.8	2	0
	Total	19			129		2.3	3	0

*O, oropharyngeal swabs; C, cloacal swabs; E, enviroment swabs. Values of brackets refer to the positive swabs detected.

Table S2. The virus strains with the highest homology.

Strains	Gene	Most homologous strain	Homology (%)	GenBank Accession Number
A/environment/Jiangxi/F0114/2022(H4N6)	PB2	A/Pink-footed goose/South Korea/19DC-44/2019 (H11N2)	99.13%	OQ296901
	PB1	A/Eurasian_Curlew/China/CZ322(7)/2019(H3N8)	98.80%	MT835198
	PA	A/Bean Goose/South Korea/KNU2021-26/2021(H6N2)	99.66%	ON564807
	HA	A/spot-billed duck/South Korea/JB32-105/2019(H4N2)	98.97%	MW494119
	NP	A/duck/Mongolia/667/2019(H3N8)	99.07%	ON514004
	NA	A/unidentified_bird/Saga/NIES889/2022(H4N6)	99.15%	LC764372
	M	A/duck/Mongolia/961/2019(H3N8)	99.21%	MT020281
	NS	A/Wild duck/South Korea/KNU2020-31/2020(H1N1)	99.49%	OK217178
A/duck/Jiangxi/F011502 /2022(H4N6)	PB2	A/Eurasian_Curlew/China/CZ322(7)/2019(H3N8)	98.85%	ON513977
	PB1	A/mallard/South Korea/JB21-22/2019(H4N6)	98.50%	MT835198
	PA	A/Bean Goose/South Korea/KNU2021-26/2021(H6N2)	99.72%	ON564807
	HA	A/spot-billed duck/South Korea/JB32-105/2019(H4N2)	99.06%	MW494119
	NP	A/duck/Mongolia/667/2019(H3N8)	99.13%	ON514004
	NA	A/duck/Bangladesh/41481/2019(H4N6)	98.23%	MW466445
	M	A/duck/Mongolia/961/2019(H3N8)	99.19%	MT020281
	NS	Wild duck/South Korea/KNU2020-31/2020(H1N1)	99.41%	OK217178
A/duck/Jiangxi/F011501 /2022(H4N6)	PB2	A/Mallard/South Korea/KNU2021-49/2021(H7N7)	98.58%	ON505898
	PB1	A/duck/Vietnam/HN5959/2019(H4N6)	98.99%	MW935118
	PA	A/wild duck/South Korea/KNU18-106/2018(H7N7)	98.59%	MN483210
	HA	A/spot-billed duck/South Korea/JB32-105/2019(H4N2)	98.73%	MW494119
	NP	A/duck/Mongolia/667/2019(H3N8)	98.65%	MT020231
	NA	A/duck/Bangladesh/41481/2019(H4N6)	98.31%	MW466445
	M	A/duck/Mongolia/961/2019(H3N8)	99.12%	MT020281
	NS	Wild duck/South Korea/KNU2020-31/2020(H1N1)	99.48	OK217178
